# Misdiagnosed Primary Pulmonary Synovial Sarcoma: A Case Report Emphasizing Diagnostic Pitfalls and the Crucial Role of Molecular Testing

**DOI:** 10.7759/cureus.79256

**Published:** 2025-02-18

**Authors:** Jiao He, Hui Chen, Ling Li Liang, Zhi Duan

**Affiliations:** 1 Department of Pathology, First Hospital of Changsha, Changsha, CHN

**Keywords:** diagnostic challenges, molecular testing, primary pulmonary synovial sarcoma, rare malignancies, ss18-ssx gene fusion

## Abstract

Primary pulmonary synovial sarcoma (PPSS) is an extremely rare malignancy that presents diagnostic challenges due to its overlapping clinical features with other lung diseases. We present the case of a 53-year-old woman with a history of bronchial artery malformation and right lung organizing pneumonia, who experienced an eight-month history of intermittent cough and sputum. Despite multiple imaging studies and biopsies at different hospitals, a definitive diagnosis was not reached until a CT scan of the chest revealed an enlarged mass in the right lower lobe. The patient underwent video-assisted thoracoscopic surgery, where intraoperative findings and pathological examination confirmed a spindle cell tumor. Immunohistochemistry and next-generation sequencing then identified the presence of a gene fusion, confirming the diagnosis of PPSS. This case illustrates how PPSS can mimic other malignancies or infections and underscores the importance of advanced diagnostic techniques, such as immunohistochemistry and molecular testing, in facilitating an accurate diagnosis.

## Introduction

Synovial sarcoma is a rare and aggressive malignant tumor of mesenchymal origin, which typically arises in soft tissues near joints. While it accounts for approximately 5-10% of all soft tissue sarcomas, its occurrence in the lung is exceedingly rare. Synovial sarcomas are mostly found in the extremities, especially near the joints, such as around the ankle, knee, or foot. Despite the term “synovial,” this type of sarcoma is not restricted to periarticular areas and can be found in various anatomical locations, including the trunk, head and neck, abdomen, pelvis, mediastinum, pleura, and even bones [1].

Primary pulmonary synovial sarcoma (PPSS) is an extremely rare tumor, accounting for fewer than 0.5% of all primary lung tumors [2]. Clinically, PPSS presents with non-specific symptoms such as cough, hemoptysis, chest pain, fever, and dyspnea, which overlap with the symptoms of other more common lung diseases. Radiologically, PPSS often presents as a poorly circumscribed lung mass that may mimic a lung infection or malignancy such as malignant mesothelioma or metastatic carcinoma. These symptoms and the non-specificity of the imaging findings result in a high rate of misdiagnosis, often delaying diagnosis until the disease is at an advanced stage. Additionally, PPSS often involves pleural invasion, leading to pleural effusion, further complicating diagnosis and treatment.

Pathologically, diagnosing PPSS can be particularly challenging due to its similarity to other tumors, such as malignant mesothelioma, pulmonary carcinomas, or even benign lesions such as granulomas. The tumor’s histological features may be non-specific [3], often showing a spindle cell morphology and requiring comprehensive immunohistochemical staining for proper classification [4]. The identification of specific genetic fusions, such as *SS18-SSX*, plays a critical role in confirming the diagnosis, as traditional diagnostic methods may be inconclusive [5].

In terms of treatment, surgical resection remains the primary approach for localized PPSS, with a focus on achieving clear margins. However, due to the rarity of PPSS, the role of adjuvant therapies, such as radiation and chemotherapy, remains controversial, and the prognosis can vary significantly depending on tumor size, metastasis, and the success of surgical intervention. The long-term survival rate for PPSS is generally lower compared to other types of lung malignancies, with patients at higher risk for recurrence, especially if there is pleural involvement or metastasis at the time of diagnosis [6].

Only a few cases of PPSS have been reported in the literature, and this case contributes to the growing body of knowledge by highlighting the challenges of pathological diagnosis, emphasizing the diagnostic challenges, and the critical role of molecular testing in confirming the diagnosis.

## Case presentation

**Figure 1:**
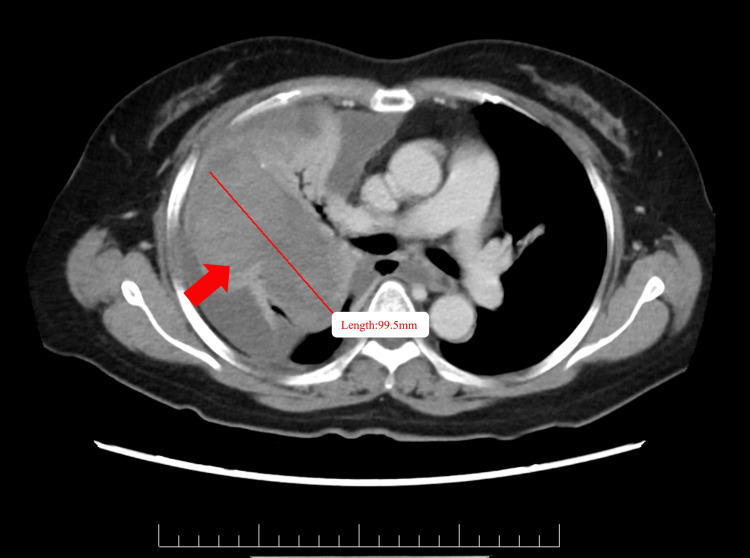
Chest CT imaging. The CT scan demonstrates a pulmonary mass in the right lower lobe, as indicated by the red arrow. The red line delineates the lesion’s maximum diameter, highlighting its extent.

A 53-year-old female with a medical history of bronchial artery malformation and right lung organizing pneumonia presented with an eight-month history of intermittent cough and sputum production. The sputum was yellowish-white, viscous, and occasionally blood-tinged, primarily occurring in the morning and evening. She denied experiencing fever, dyspnea, night sweats, or weight loss. Despite visits to different hospitals, imaging studies revealed an indeterminate mass in the right lower lobe, and multiple pulmonary biopsies showed no definitive abnormalities (Figure 1). Symptom-directed treatments, including anti-infective therapy and cough relief, proved ineffective (Table 1).

**Table 1:** Patient treatment timeline. This table outlines the chronological sequence of key diagnostic assessments, therapeutic interventions, and follow-up measures for the patient.

Date	Event/Hospital visit	Key findings/Diagnostic tests	Interventions/Treatments
January 2024	Initial onset of symptoms	Patient (a 53-year-old female) experienced cough and sputum production	No specific treatment details available
Multiple visits, up to September 4, 2024	Hospital A	Chest enhanced CT scan: right lower lobe mass with increased density, suspected tumor or infection. Biopsy via percutaneous puncture: pathological diagnosis of organizing pneumonia. Recent imaging on September 4: mass enlargement, increased infectious lesions	Repeated anti-infective treatments and symptomatic care. Chest puncture drew ~300 mL pleural effusion (on September 5)
September 5, 2024	Transfer to Hospital B	Patient self-referred due to persistent fever, chest pain, dyspnea, and cough. Admitted to the Cardiothoracic Surgery Unit	Further evaluation indicated a suspected ruptured tumor. Planned for surgical intervention
September 15, 2024	Surgical treatment	Intraoperative findings: tumor with necrosis in the right lower lobe. Pathology: monophasic synovial sarcoma (*SS18-SSX2* gene fusion).	Extended right lower lobectomy, postoperative supportive care
Postoperative	Follow-up	Stable condition at the three-month follow-up. No evidence of recurrence	No additional adjuvant therapy reported at this stage

In September 2024, the patient presented with a one-week history of intermittent chest pain, dyspnea, and fever. A chest CT scan revealed that the right lower lobe mass had increased in size compared to prior imaging, showing an irregular soft tissue density shadow. While the mass had a well-defined border, the contrast-enhanced scan demonstrated uneven enhancement. Additionally, the surrounding lung tissue and right pulmonary artery were compressed, with some bronchial occlusion observed. Pleural effusion and progression of infectious lesions were noted. Laboratory tests indicated severe anemia and a slight elevation in neuron-specific enolase levels, with other tumor markers within normal limits. Bronchoscopy showed compression and edema of the right middle lobe bronchus; however, lavage cytology was negative for malignancy. Given the persistence of symptoms and the inconclusive findings from previous examinations, the decision was made to perform video-assisted thoracoscopic surgery (VATS).

Intraoperative findings included extensive pleural adhesions, intracapsular hemorrhagic-purulent fluid, and fibrotic deposits. The right lower lobe contained a 60-mm cystic lesion filled with necrotic tissue and purulent debris. Because of bronchial invasion, the surgeon performed an extended right lower lobectomy and sampled nearby lymph nodes. The resected specimen measured 90 × 50 × 30 mm and was cystic with yellow-gray necrotic tissue.

**Figure 2:**
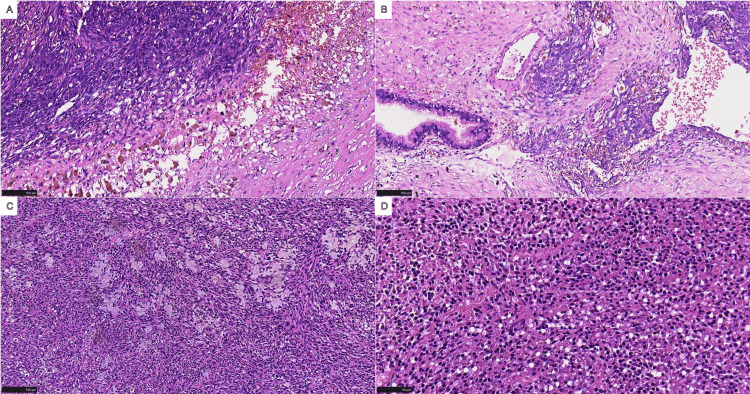
Histopathological findings. Microscopically, the tumor is characterized by a monophasic spindle cell neoplasm, with cells predominantly arranged in sheets and bundles and accompanied by focal areas of hyalinization.

Microscopic examination revealed a spindle cell tumor with eosinophilic cytoplasm, hyperchromatic nuclei, frequent mitotic figures, focal necrosis, and a hemangiopericytoma-like vascular pattern (Figure 2). Based on these findings, we suspected that the tumor originated from soft tissue and was non-epithelial in nature. To further differentiate the tumor from other malignancies, such as leiomyosarcoma, dedifferentiated liposarcoma, malignant fibrous histiocytoma, and malignant stromal tumors, immunohistochemistry was performed.

**Figure 3:**
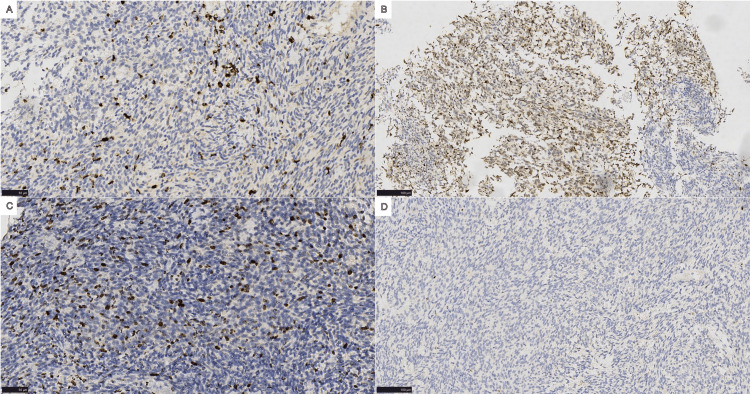
Immunohistochemical expression. (A) Vimentin, (B) pan-cytokeratin), (C) Ki-67 (antigen identified by monoclonal antibody Ki-67), and (D) cluster of differentiation 34.

**Figure 4:**
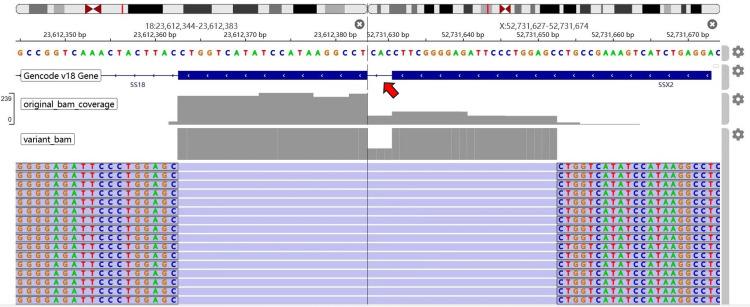
Schematic representation of the SS18-SSX2 gene fusion. The *SS18-SSX2* rearrangement produces a fusion gene that retains the promoter and exons up to exon 10 of the *SS18* gene at the 5′ end, and exons 5 through the terminal exon of the *SSX2* gene at the 3′ end. This fusion event is predicted to generate an out-of-frame transcript, which may significantly alter the function of the resulting chimeric protein.

The analysis showed positive staining for vimentin, Ki-67 (antigen identified by monoclonal antibody Ki-67, ~25%), pan-cytokeratin, epithelial membrane antigen), and β-catenin, while other markers were negative (Figure 3). To confirm the diagnosis, next-generation sequencing (NGS) was employed, which identified the *SS18-SSX2* (synovial sarcoma) gene fusion (Figure 4), a molecular hallmark of synovial sarcoma. The final diagnosis was PPSS, spindle cell type, with bronchial lymph node metastasis. No tumor deposits were found in any of the 11 lymph node groups sampled. The patient recovered well postoperatively and did not receive any further treatment. At the three-month follow-up, the patient remained stable, with no recurrence or metastasis.

## Discussion

PPSS is a rare and aggressive tumor that often presents significant diagnostic challenges due to its non-specific clinical features and radiological appearance. The clinical presentation of PPSS overlaps with many other pulmonary conditions, including infections and more common malignancies, which can easily lead to misdiagnosis. In this case, the patient’s initial presentation of persistent cough, hemoptysis, and chest pain, along with radiological findings that suggested a mass in the lung with poorly defined borders, led to a misdiagnosis of organizing pneumonia at first. This was further compounded by the presence of cystic changes and abscess formation, which are typically associated with infectious processes rather than neoplastic diseases.

Misdiagnosis in PPSS is not uncommon. In fact, several studies have reported similar diagnostic pitfalls. For instance, in a study by Wu et al. [7], PPSS was initially mistaken for malignant mesothelioma, with both sharing features such as pleural involvement and ill-defined masses. Additionally, benign lesions such as hamartomas and inflammatory granulomas have also been mistaken for PPSS, as reported by Kim et al. [8]. These benign lesions often present with similar radiological features, such as peripheral masses and calcifications, which are commonly associated with non-cancerous growths, further complicating the diagnostic process. Notably, calcification within pulmonary nodules has been identified as a distinguishing feature of benignity in many cases, but it has also been observed in PPSS, adding another layer of diagnostic confusion [8-10].

In our case, the presence of pleural effusion and infectious lesions on imaging further contributed to the initial misdiagnosis of a lung infection. Despite multiple biopsies, the initial pathological findings were inconclusive, with organizing pneumonia being the initial diagnosis. It was only after a more thorough diagnostic workup, including thoracoscopic exploration and molecular testing (*SS18-SSX2* gene fusion), that a definitive diagnosis of PPSS was made.

This case underscores the importance of a comprehensive diagnostic approach, especially in rare malignancies such as PPSS. Molecular testing, such as the identification of the *SS18-SSX2* fusion gene, has proven to be a valuable tool in confirming the diagnosis, distinguishing PPSS from other sarcomas and benign lesions with overlapping features [11]. The sensitivity and specificity of molecular techniques, especially NGS, offer a high degree of diagnostic confidence, which can prevent delays in diagnosis and ensure timely treatment [12].

In summary, the diagnostic challenges in PPSS arise not only from its clinical and radiological features but also from its ability to mimic a variety of other diseases, both malignant and benign. Increasing awareness of these diagnostic pitfalls, combined with the use of advanced molecular techniques, will aid clinicians in more accurately identifying PPSS and improving patient outcomes.

## Conclusions

PPSS is an extremely rare and highly aggressive malignancy that poses significant challenges in both diagnosis and treatment. The non-specific clinical features and radiological findings, along with the possibility of pleural invasion, often lead to misdiagnosis. In this case, despite multiple thoracic biopsies, the diagnosis was ultimately confirmed through thoracoscopic exploration and molecular testing that identified the *SS18-SSX2* gene fusion. This case highlights the importance of integrating advanced diagnostic methods, including histopathology, immunohistochemistry, and molecular testing, to achieve an accurate diagnosis.

Molecular testing, such as the identification of the *SS18-SSX2* gene fusion, plays a crucial role in differentiating PPSS from other malignancies and benign conditions. Advanced techniques such as NGS provide high diagnostic accuracy and should be incorporated into routine diagnostic workflows, especially in cases where the diagnosis remains unclear. Furthermore, this case underscores the importance of surgical biopsy when faced with a challenging diagnosis, as it is a crucial step in confirming the diagnosis and guiding subsequent treatment decisions.

In conclusion, this case emphasizes the need for heightened awareness of PPSS in clinical practice, the importance of integrating molecular diagnostics into the diagnostic workflow, and the value of surgical biopsy in cases where non-invasive tests fail to provide conclusive results.
